# The clinicopathological significances and related signal pathways of BTG3 mRNA expression in cancers: A bioinformatics analysis

**DOI:** 10.3389/fgene.2022.1006582

**Published:** 2022-09-16

**Authors:** Hua-Chuan Zheng, Hang Xue, Cong-Yu Zhang, Kai-Hang Shi, Rui Zhang

**Affiliations:** ^1^ Department of Oncology, The Affiliated Hospital of Chengde Medical University, Chengde, China; ^2^ Cancer Center, The First Affiliated Hospital of Jinzhou Medical University, Jinzhou, China; ^3^ Department of Dermatology, The Affiliated Hospital of Chengde Medical University, Chengde, China; ^4^ Department of Colorectal Surgery, Liaoning Cancer Hospital, Shenyang, China

**Keywords:** *BTG3*, bioinformatics analysis, carcinogenesis, aggressive behavior, prognosis

## Abstract

B cell transposition gene 3 (BTG3) is reported to be a tumor suppressor and suppresses proliferation and cell cycle progression. This study aims to analyze the clinicopathological and prognostic significances, and signal pathways of *BTG3* mRNA expression in human beings through bioinformatics analysis. We analyzed *BTG3* expression using Oncomine, TCGA (the cancer genome atlas), Xiantao, UALCAN (The University of ALabama at Birmingham Cancer data analysis Portal) and Kaplan-Meier plotter databases. Down-regulated *BTG3* expression was observed in lung and breast cancers, compared with normal tissues (*p* < 0.05), but not for gastric and ovarian cancer (*p* < 0.05). The methylation of *BTG3* was shown to be adversely correlated with its mRNA expression (*p* < 0.05). *BTG3* expression was higher in gastric intestinal-type than diffuse-type carcinomas, G_1_ than G_3_ carcinomas (*p* < 0.05), in female than male cancer patients, T_1-2_ than T_3-4_, and adenocarcinoma than squamous cell carcinoma of lung cancer (*p* < 0.05), in invasive ductal than lobular carcinoma, N_0_ than N_1_ and N_3_, TNBC (triple-negative breast cancer) than luminal and Her2+, and Her2+ than luminal cancer of breast cancer (*p* < 0.05), and G_3_ than G_2_ ovarian carcinoma (*p* < 0.05). *BTG3* expression was positively related to the survival rate of gastric and ovarian cancer patients (*p* < 0.05), but not for breast cancer (*p* < 0.05). KEGG and PPI (protein-protein interaction) analysis showed that the *BTG3* was involved in cell cycle and DNA replication, digestion and absorption of fat and protein, spliceosome and ribosome in cancer. *BTG3* expression was positively linked to carcinogenesis, histogenesis, and aggressive behaviors, and was employed to evaluate the prognosis of cancers by regulating cell cycle, metabolism, splicing and translation of RNA.

## Introduction

B cell transposition gene 3 (BTG3) belongs to a member of B-cell translocation gene family, and maps to human chromosome 21q21.1 (Guéhenneux et al., 1997). Its encoding protein has been reported to be a tumor suppressor in some malignancies, including gastric cancer, breast cancer, renal cell carcinoma, esophageal adenocarcinoma, hepatocellular carcinoma, lung cancer, ovarian cancer, and prostate cancer (Yu et al., 2008; Majid et al., 2009; Lin et al., 2012; Chen et al., 2013; Deng et al., 2013; Lv et al., 2013; Du et al., 2015; Gou et al., 2015; Ren et al., 2015). In the nuclear compartment, BTG3 protein can interact with E2F1 (E2F transcription factor 1), Smad8 receptor-regulated Smad transcription factor, and CCR4 (C-C motif chemokine receptor 4) transcription factor-associated protein Caf1 to suppress finally cell proliferation and cell cycle progression (Yoshida et al., 2001; Ou et al., 2007; Miyai et al., 2009). BTG3 maintains genomic stability by promoting Lys63-linked ubiquitination and CHK1 (checkpoint kinase 1) activation, whereas BTG3 can be phosphorylated and activated *via* its interaction with CHK1 as a positive feedback loop (Cheng et al., 2013). In the cytosolic compartment, BTG3 binds to and suppresses src, Akt and Ras/MAP kinase signaling (Rahmani, 2006; Cheng et al., 2015). Ma et al. (2020) demonstrated that the combination of hypoxia and BTG3 expression could induce radiation resistance, indicating an important role of BTG3 in hypoxia-induced radiation resistance of colorectal cancer cells. Cucurbitacin B inhibited cell proliferation and anti-apoptosis of colorectal cancer by the reactivation of BTG3 by promoter demethylation (Mao et al., 2019).

By postnatal 21 months, BTG3-deficiency might promote bone morphogenetic protein-induced ectopic bone formation and lung adenocarcinogenesis (Yoneda et al., 2009). BTG3 deficiency triggers acute cellular senescence *via* the Erk-AP-1-JMJD3-p16 pathway (Lin et al., 2012). MiR-142-5p strengthens cell growth and migration in renal cell carcinoma, while miR-93 desensitizes esophageal cancer to radiotherapy by targeting BTG3 (Cui et al., 2017; Liu et al., 2017). IASPP promotes miR-20a expression that restores cell invasion and cisplatin chemoresistance of cervical cancer cells by targeting BTG3 (Xiong et al., 2017). A body of evidence identified BTG3 as a direct downstream target of miR-519c-3p and miR-20b-5p, which promoted proliferation and migration in hepatocellular carcinoma and colorectal cancer cells, respectively (Peng et al., 2019; Wang et al., 2019). Enkhnaran et al. (2022) demonstrated that miR-106b-5p promoted cell proliferation and cell cycle and increased hepatocellular carcinoma cells’ resistance to sorafenib through the BTG3/Bcl-xL/p27 signaling pathway.

In our previous work, BTG3 overexpression was demonstrated to reverse the aggressive phenotypes of gastric and colorectal cancer cells (Gou et al., 2015; Zheng et al., 2017). Here, we aimed to clarify the clinicopathological and prognostic significances, and related signal pathways of *BTG3* mRNA expression in cancers by a bioinformatics analysis.

## Material and methods

### Oncomine database analysis

The individual gene expression level of *BTG3* mRNA was analyzed using Oncomine (www.oncomine.org), a cancer microarray database and web-based data mining platform for a new discovery from genome-wide expression analyses. We compared the differences in *BTG3* mRNA levels between normal tissue and cancer. All data were log-transformed, the median centered per array, and the standard deviation normalized to one per array.

### The cancer genome atlas (TCGA) database analysis

The expression data (RNA-seqV2) and clinicopathological data of gastric (*n* = 392), lung (*n* = 865), breast (*n* = 1,093), and ovarian (*n* = 304) cancer patients were downloaded from the TCGA database (https://cancergenome.nih.gov/abouttcga/overview) by TCGA-assembler in R software. We integrated the raw data, analyzed *BTG3* mRNA expression in the cancers, and compared it with clinicopathological and prognostic data of cancer patients. The means were compared with student *t*-test. Kaplan-Meier survival plots were generated with survival curves and compared by the log-rank statistic. Cox’s proportional hazards model was employed for multivariate analysis. SPSS 17.0 software was employed to analyze all data. Two-sided *p* < 0.05 was considered statistically significant.

### Kaplan-Meier (KM) plotter analysis

The prognostic significance of ***BTG3*** mRNA was also analyzed in gastric, lung, breast, and ovarian cancers using KM plotter (https://kmplot.com/analysis/).

### The university of aLabama at birmingham cancer data analysis portal analysis

The expression and methylation of *BTG3* gene were analyzed using the UALCAN database (http://ualcan.path.uab.edu/). They were also compared with the clinicopathological and prognostic features of gastric, lung, breast, and ovarian cancers.

### Xiantao analysis

The expression and methylation of *BTG3* gene were analyzed using the xiantao platform (https://www.xiantao.love/). Additionally, we discovered the differential and related genes using Xiantao. The differential genes were used to build the PPI (protein-protein interaction) network and identify the important hub genes. These genes were submitted to KEGG (Kyoto Encyclopedia of Genes and Genomes) analysis in order to build signal pathways.

## Results

### The clinicopathological and prognostic significances of *BTG3* mRNA expression in gastric cancer

**FIGURE 1:**
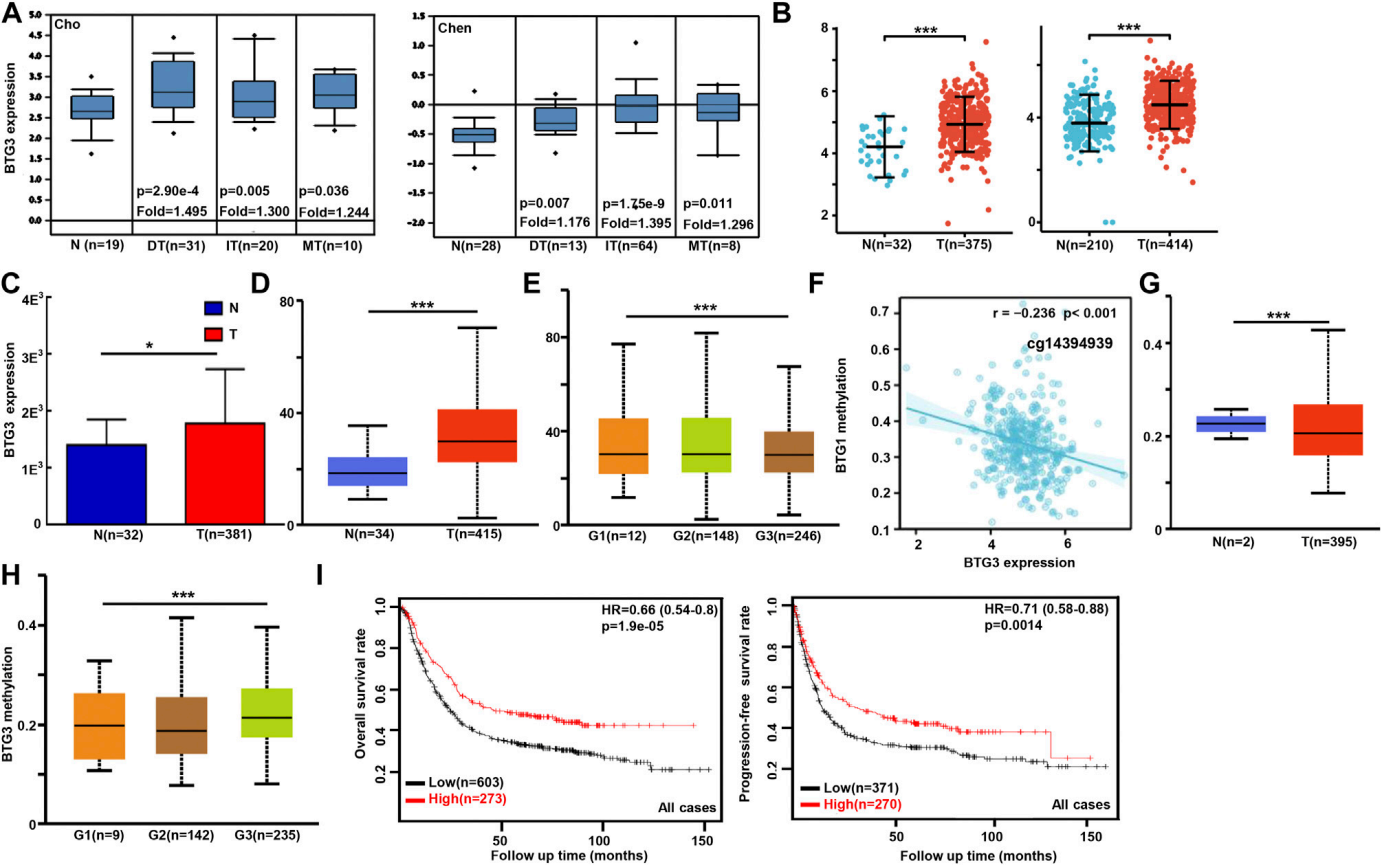
The clinicopathological and prognostic significances of *BTG3* mRNA expression in gastric cancer Cho’s and Chen’s datasets were used for bioinformatics analysis to explore *BTG3* expression in gastric cancer. A higher *BTG3* expression was detectable in gastric cancer than that in normal mucosa, even stratified into intestinal- (IT), diffuse- (DT), and mixed-type (MT) carcinomas by Lauren’s classification [**(A)**, *p* < 0.05], in line with the findings from Xiantao **(B)**, TCGA **(C)** and UALCAN **(D)** databases (*p* < 0.05). UALCAN database showed that *BTG3* was more expressed in G_1_ than G_3_ carcinomas [**(E)**, *p* < 0.05]. The negative relationship between *BTG3* mRNA expression and methylation was found in gastric cancer using Xiantao database **(F)**. Its methylation was lower in gastric cancer than normal tissues in UALCAN **(G)**. We also compared *BTG3* methylation with histological grading of gastric cancer **(H)**. According to the data from Kaplan-Meier plotter, *BTG3* expression was positively related to both overall and progression-free survival rates of the patients with gastric cancer [**(I)**, *p* < 0.05)]. Note: N, normal; T, tumor; HR, hazard ratio.

We used Cho’s and Chen’s datasets to perform bioinformatics analysis, and found that *BTG3* expression was higher in gastric cancer than in normal tissues, even stratified into intestinal-, diffuse-, and mixed-type carcinomas (Figure 1A, *p* < 0.05). In TCGA (Figure 1B), Xiantao (Figure 1C) and UALCAN (Figure 1D) data, it was the same for *BTG3* expression (*p* < 0.05). *BTG3* expression was higher in G_1_ than G_3_ carcinomas by UALCAN (Figure 1E, *p* < 0.05). There was a negative correlation between *BTG3* mRNA and methylation (cg23273752, cg12602426, cg01168851, cg04464940, cg14394939, and cg08875503) by Xiantao (Figure 1F, *p* < 0.05). *BTG3* methylation was lower in gastric cancer than in normal tissues (Figure 1G, *p* < 0.05), and G_1_ than G_3_ carcinoma (Figure 1H, *p* < 0.05) by UALCAN. According to Kaplan-Meier plotter, we found that a higher *BTG3* expression was positively correlated with overall and progression-free survival rates of all cancer patients, even stratified by gender and Her2+ expression (Figure 1I and Table 1, *p* < 0.05). The overall survival rate of the patients with intestinal-, diffuse-, or mixed-type carcinoma was higher in *BTG3* overexpression than in underexpression groups (Table 1, *p* < 0.05). There appeared to be a positive relationship between *BTG*3 expression and the overall survival rate of the patients with 5-FU-based adjuvant (Table 1, *p* < 0.05). It was the same for the progression-free survival in the patients with poorly-differentiated adenocarcinoma, 5-FU-based or other adjuvant treatment (Table 1, *p* < 0.05).

**TABLE 1:** The prognostic significance of *BTG3* mRNA in gastric cancer.

Clinicopathological features	Overall survival		Progression-free survival	
	Hazard ratio	*p*	Hazard ratio	*p*
Sex				
Female	0.62 (0.41–0.93)	0.019	0.64 (0.41–0.99)	0.046
Male	0.64 (0.51–0.81)	0.00014	0.68 (0.53–0.87)	0.0018
T				
2	0.61 (0.38–0.97)	0.035	0.63 (0.38–1.03)	0.062
3	0.79 (0.53–1.17)	0.24	0.84 (0.59–1.2)	0.33
4	2.21 (0.94–5.17)	0.061	2.03 (0.93–4.41)	0.069
N				
0	2.22 (0.75–6.61)	0.14	2.23 (0.74–6.65)	0.14
1–3	0.71 (0.54–0.93)	0.012	0.8 (0.61–1.03)	0.081
1	0.69 (0.46–1.05)	0.079	0.68 (0.42–1.09)	0.1
2	0.7 (0.44–1.12)	0.14	0.76 (0.5–1.17)	0.21
3	2.07 (1.16–3.71)	0.013	1.58 (0.93–2.68)	0.088
M				
0	0.76 (0.57–1.02)	0.066	1.22 (0.91–1.64)	0.18
1	0.55 (0.29–1.06)	0.07		
TNM staging				
I	0.34 (0.11–1.08)	0.057	0.48 (0.16–1.45)	0.18
II	0.58 (0.28–1.17)	0.12	0.5 (0.23–1.09)	0.076
III	0.76 (0.56–1.03)	0.08	1.33 (0.88–2.01)	0.18
IV	0.71 (0.47–1.08)	0.11	1.23 (0.84–1.8)	0.29
Differentiation				
Well-differentiated	—	—	—	—
Moderately-differentiated	1.35 (0.68–2.69)	0.39	1.42 (0.75–2.67)	0.28
Poorly-differentiated	1.25 (0.81–1.92)	0.31	1.61 (1.02–2.54)	0.039
Lauren’s classification				
Intestinal-type	0.66 (0.47–0.92)	0.013	0.73 (0.52–1.04)	0.084
Diffuse-type	0.62 (0.41–0.94)	0.022	0.7 (0.47–1.04)	0.073
Mixed-type	0.23 (0.06–0.81)	0.013	0.57 (0.2–1.59)	0.28
Her2 positivity				
−	0.63 (0.5–0.78)	4e−05	0.6 (0.45–0.81)	0.00058
+	1.31 (1.01–1.71)	0.043	1.51 (1.09–2.08)	0.012
Perforation				
—	1.38 (0.91–2.09)	0.12	1.42 (0.97–2.08)	0.07
Treatment				
Surgery alone	0.74 (0.54–1.02)	0.066	1.36 (1–1.87)	0.053
5-FU-based adjuvant	1.59 (1.08–2.35)	0.018	1.53 (1.08–2.17)	0.016
Other adjuvant	3.56 (0.82–15.42)	0.07	2.6 (1.17–5.78)	0.015

### The clinicopathological and prognostic significances of *BTG3* mRNA expression in lung cancer

**FIGURE 2:**
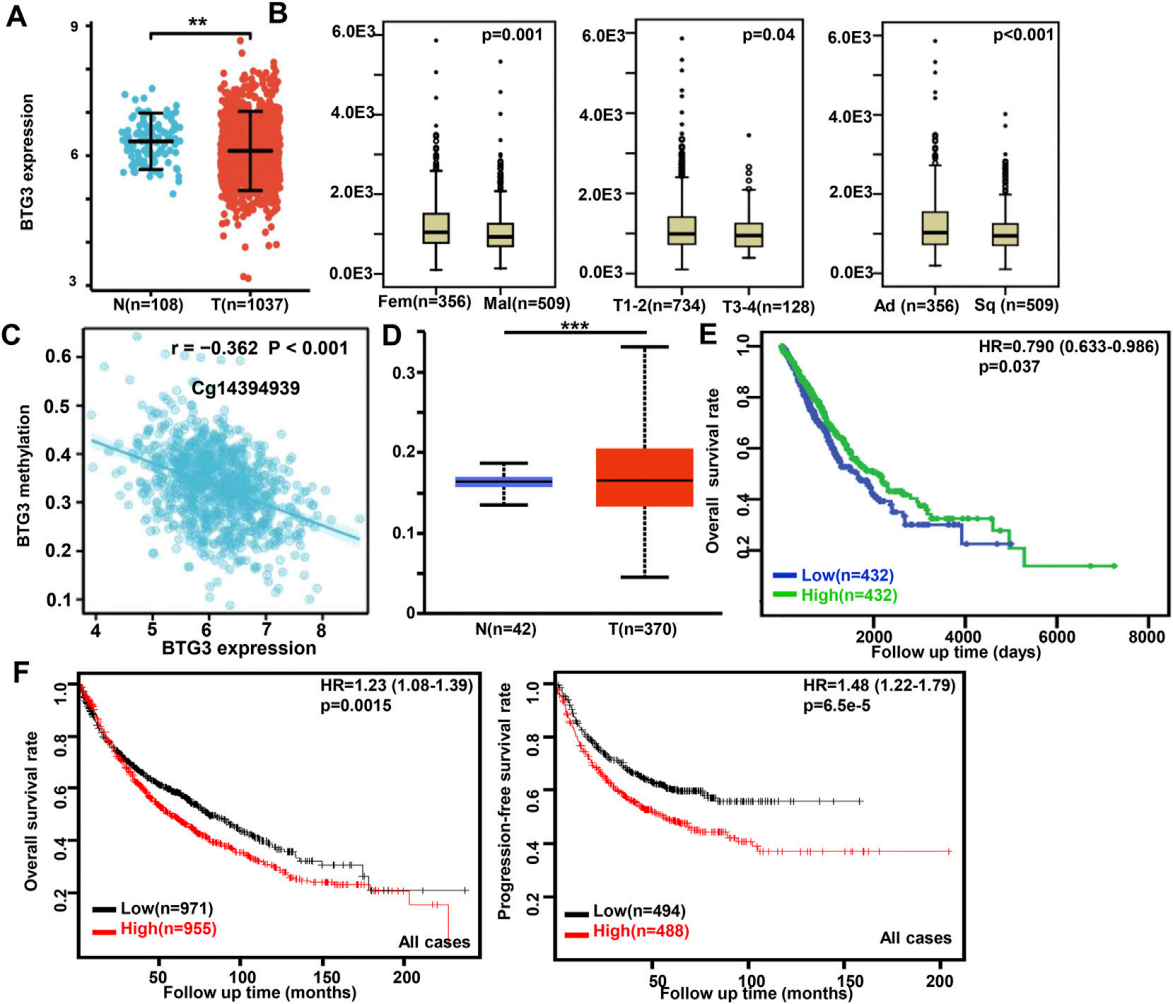
The clinicopathological and prognostic significances of *BTG3* mRNA expression in lung cancer Xiantao dataset was employed for bioinformatics analysis to analyze *BTG3* expression during lung carcinogenesis **(A)**. *BTG3* expression was compared with gender, histological subtyping, and T staging of the cancer patients by TCGA database **(B)**. The negative relationship between *BTG3* mRNA expression and methylation was analyzed in lung cancer using Xiantao database **(C)**. Its methylation was higher in lung cancer than normal tissues in UALCAN **(D)**. The correlation between *BTG3* expression and overall or post-progression survival rate of the patients with lung cancer was analyzed using TCGA database **(E)** and Kaplan-Meier plotter **(F)**. Note: N, normal; T, tumor; Ad, adenocarcinoma; Sq, squamous cell carcinoma; HR, hazard ratio.

In Xiantao, *BTG3* expression was lower in lung cancer than in normal tissues (Figure 2A, *p* < 0.05). In TCGA database, BTG3 expression was higher in female than male cancer patients, T_1-2_ than T_3-4_, and adenocarcinoma than squamous cell carcinoma patients (Figure 2B, *p* < 0.05). There was a negative correlation between *BTG3* mRNA and its methylation (cg14380517, cg10696191, cg03232933, cg05762769, cg14394939, and cg03232933) by xiantao (Figure 2C, *p* < 0.05). *BTG3* methylation was higher in lung cancer than in normal tissues (Figure 2D, *p* < 0.05). In TCGA, *BTG3* expression was positively associated with a high overall survival rate of cancer patients (Figure 2E, *p* < 0.05). Cox’s risk proportional regression model indicated that T staging, lymph node status and *BTG3* expression were independent prognostic factors for lung cancer patients (Table 2, *p* < 0.05). According to Kaplan-Meier plotter, we found that a higher *BTG3* expression was negatively correlated with overall survival rates of all cancer patients, female or male patients, adenocarcinoma patients, N_1_, M_0_, Stage I and II cancer patients, smoking and non-smoking patients, or those with surgical margin negative (Figure 2F, *p* < 0.05). All, female T_1_, T_2_, or N_1_ cancer patients with high *BTG3* expression showed a short progression-free survival time than those with its low expression (*p* < 0.05, data not shown). There appeared to be a negative relationship between *BTG3* expression and the progression-free survival rate of cancer patients with surgical margin negative or smoking cancer patients (*p* < 0.05, data not shown).

**TABLE 2:** Multivariate analysis of hazard factors of the prognosis of the patients with lung cancer.

Clinicopathological features	Hazard ratio (95% CI)	*p*
Gender (Female/male)	1.088 (0.853–1.387)	0.496
Stage T (T1-2/T3-4)	1.525 (1.089–2.137)	0.014
Lymph node status (−/+)	1.591 (1.223–2.070)	0.001
TNM staging (I-II/III-IV)	1.154 (0.817–1.631)	0.415
Histological classification (Ad/Sq)	0.996 (0.787–1.260)	0.971
*BTG3* mRNA expression (low/high)	0.795 (0.634–0.997)	0.047

### The clinicopathological and prognostic significances of *BTG3* mRNA expression in breast cancer

**FIGURE 3:**
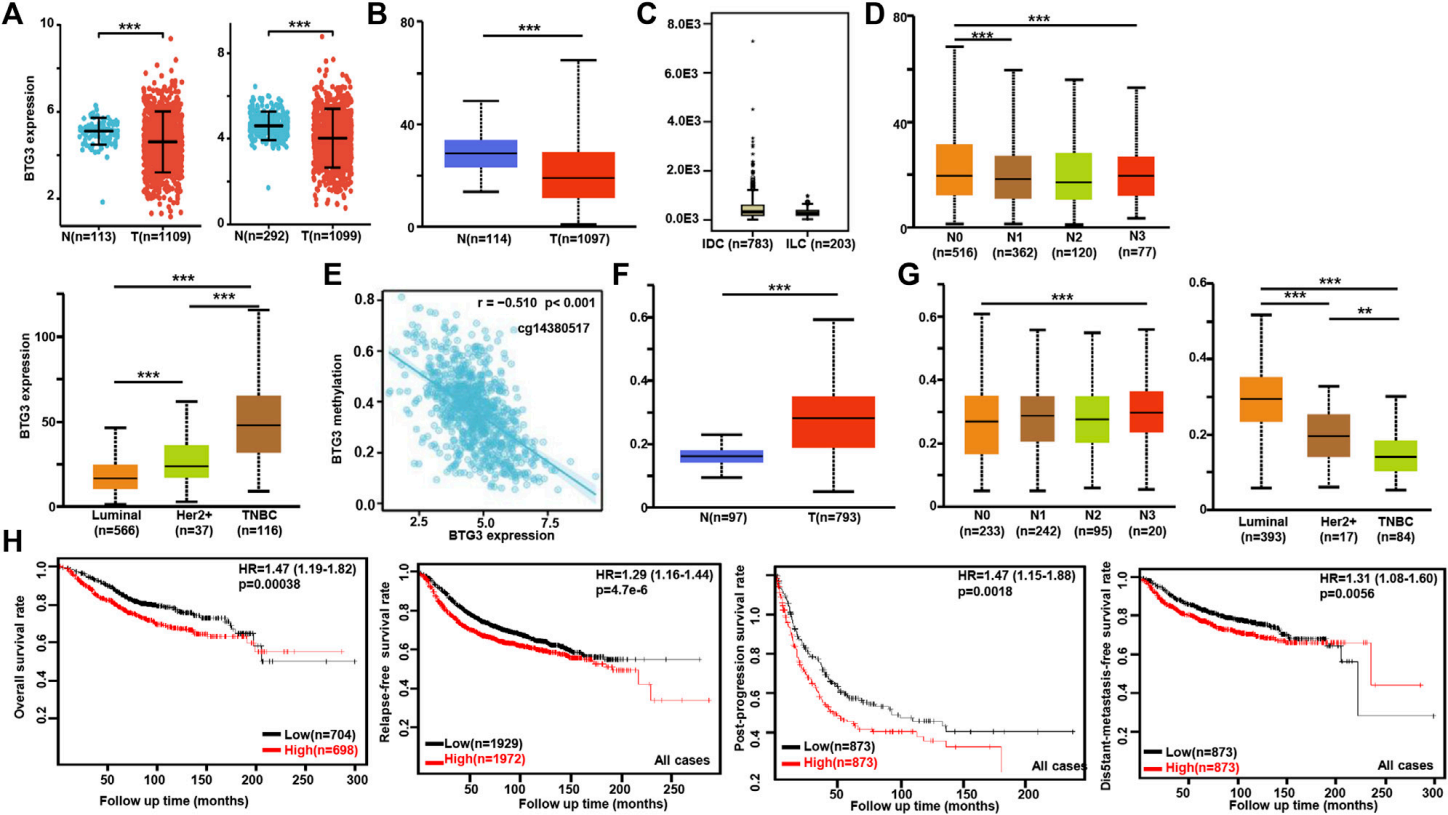
The clinicopathological and prognostic significances of *BTG3* mRNA expression in breast cancer. According to Xiantao **(A)** and UALCAN **(B)** databases, *BTG3* hypoexpression was detectable in breast cancer, compared with breast normal tissue (*p* < 0.05). TCGA database showed a higher *BTG3* expression in invasive ductal (IDC) than lobular (ILC) carcinoma [**(C)**, *p* < 0.05]. UALCAN database was also used to compare *BTG3* expression with N stage and molecular subtyping of breast cancer **(D)**. The negative relationship between *BTG3* mRNA expression and methylation was analyzed in breast cancer using Xiantao database **(E)**. Its methylation was higher in breast cancer than normal tissues **(F)**, and compared with N stage and molecular subtyping **(G)** in UALCAN. The correlation between *BTG3* expression and overall, post-progression, distant-metastasis-free or relapse-free survival rate was analyzed in the patients with breast cancer using Kaplan-Meier plotter **(H)**. Note: N, normal tissue; T, tumor; TNBC, triple-negative breast cancer; HR, hazard ratio.

According to Xiantao (Figure 3A) and UALCAN (Figure 3B) databases, we found that *BTG3* expression was lower in breast cancer than in normal tissues (*p* < 0.05). TCGA database showed a higher *BTG3* expression in invasive ductal than lobular carcinoma (Figure 3C, *p* < 0.05). It was higher in N_0_ than N_1_ and N_3_, TNBC (triple-negative breast cancer) than luminal and Her2+, and Her2+ than Luminal cancer patients by UALCAN (Figure 3D, *p* < 0.05). As summarized in Table 3, BTG3 mRNA expression was negatively associated with elder age, non-Asian race, non-TNBC, ER positivity, and PR positivity (*p* < 0.05). There was a negative correlation between *BTG3* mRNA and its methylation (cg14380517, cg10696191, cg03232933, cg05762769, cg27075724, cg02652260, cg23273752, cg12602426, cg01168851, cg20227212, cg04464940, and cg14394939) by Xiantao (Figure 3E, *p* < 0.05). *BTG3* methylation was higher in breast cancer than normal tissues (Figure 3F, *p* < 0.05), N_3_ than N_0_, Luminal and Her2+ than TNBC, Her2+ than Luminal cancer patients by UALCAN (Figure 3G, *p* < 0.05). According to Kaplan-Meier plotter, we found that a higher *BTG3* expression was negatively correlated with overall, progression-free, post-progression, and distant-metastasis-free survival rates of all cancer patients (Figure 3H, *p* < 0.05). There appeared to be a negative relationship between *BTG3* expression and the overall survival rate of patients with Luminal-B breast cancer (*p* < 0.05, data not shown). The relapse-free survival rate of the cancer patient with or without lymph node metastasis was lower in the groups of high *BTG3* expression than its low expression (*p* < 0.05, data not shown). A negative association between *BTG3* expression and relapse-free prognosis was observed in Luminal-B cancer patients (*p* < 0.05, data not shown). ER (estrogen receptor)- positive or Her2-negative cancer patients with high *BTG3* expression showed a shorter overall survival time than those with its low expression (*p* < 0.05, data not shown).

**TABLE 3:** The correlation between *BTG3* mRNA expression and clinicopathological characteristics of breast cancer.

Characteravbistic	Variables	Low expression	High expression	*p*
Age, *n* (%)	≤60	262 (24.2%)	339 (31.3%)	<0.001
	>60	279 (25.8%)	203 (18.7%)	
Race, *n* (%)	Asian	34 (3.4%)	26 (2.6%)	0.009
	Black or African American	72 (7.2%)	109 (11%)	
	White	389 (39.1%)	364 (36.6%)	
PR status, *n* (%)	Negative	104 (10.1%)	238 (23%)	<0.001
	Indeterminate	3 (0.3%)	1 (0.1%)	
	Positive	410 (39.7%)	278 (26.9%)	
ER status, *n* (%)	Negative	39 (3.8%)	201 (19.4%)	<0.001
	Indeterminate	1 (0.1%)	1 (0.1%)	
	Positive	477 (46.1%)	316 (30.5%)	
PAM50, *n* (%)	Normal	11 (1%)	29 (2.7%)	<0.001
	LumA	376 (34.7%)	186 (17.2%)	
	LumB	115 (10.6%)	89 (8.2%)	
	Her2	29 (2.7%)	53 (4.9%)	
	Basal	10 (0.9%)	185 (17.1%)	

ER, estrogen receptor; PR, progesterone receptor.

### The clinicopathological and prognostic significances of *BTG3* mRNA expression in ovarian cancer

**FIGURE 4:**
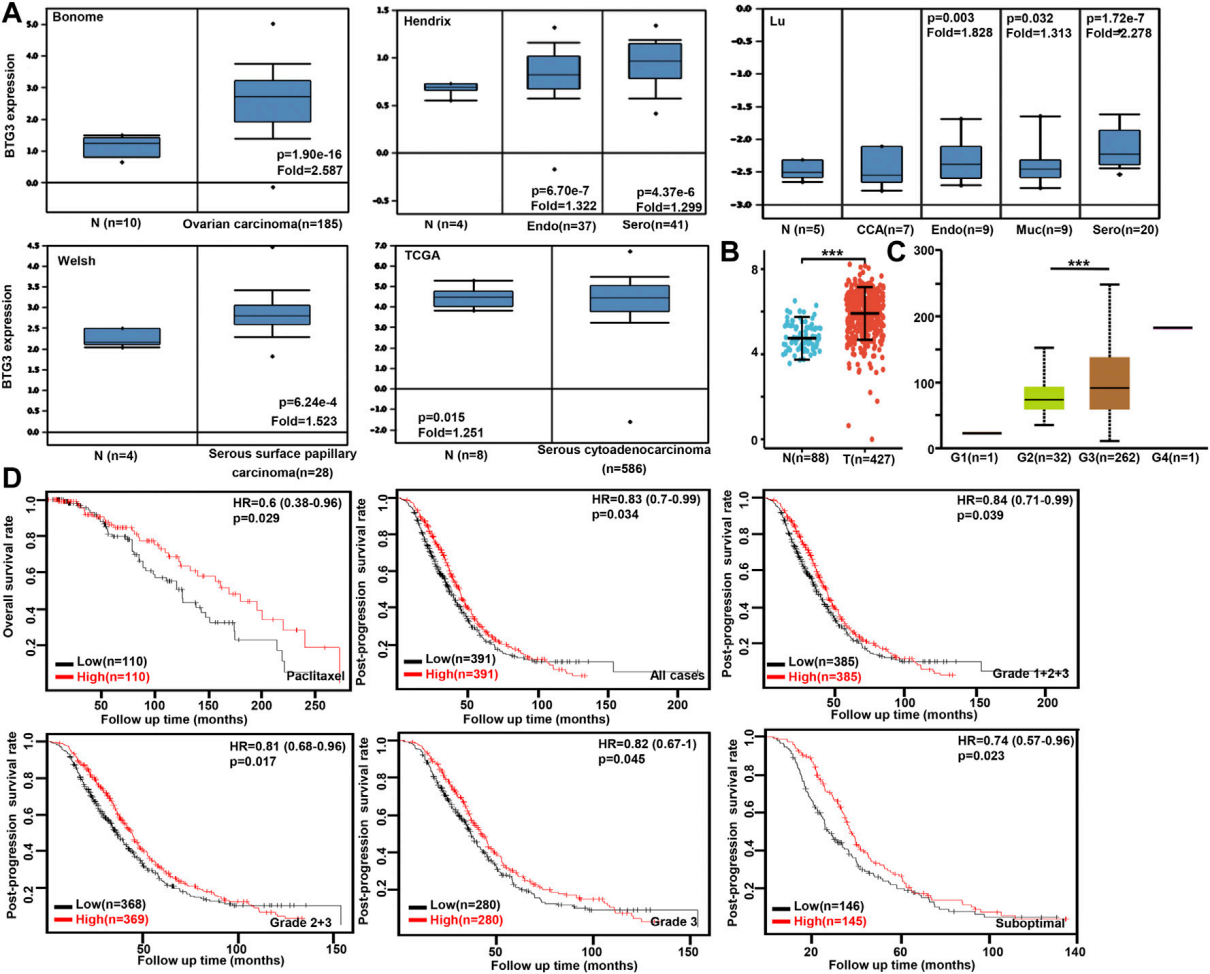
The clinicopathological and prognostic significances of *BTG3* mRNA expression in ovarian cancer Oncomine **(A)** and Xiantao **(B)** datasets were employed for bioinformatics analysis to observe BTG3 expression in ovarian cancer. A lower *BTG3* expression was detectable in ovary than that in ovarian carcinoma, clear cell adenocarcinoma (CCA), endometriod (Endo), mucinous (Muc) and serous (Sero) adenocarcinoma (*p* < 0.05). *BTG3* expression was compared with histological grading of ovarian cancer **(C)**. The correlation between *BTG3* expression and overall, or post-progression survival rate was analyzed in the patients with ovarian cancer using Kaplan-Meier plotter, even stratified by different clinicopathological parameters [**(D)**, *p* < 0.05]. Note: N, normal tissue; T, tumor; HR, hazard ratio.

We performed bioinformatics analysis of *BTG3* expression in ovarian cancer using Bonome’s, Hendrix’s, Lu’s, Welsh’s, and TCGA’s datasets. *BTG3* expression was higher in ovarian cancer than normal mucosa regardless of histological subtyping (Figure 4A, *p* < 0.05). It was the same for Xiantao data (Figure 4B, *p* < 0.05). *BTG3* expression was higher in G_3_ than G_2_ carcinoma patients by UALCAN (Figure 4C, *p* < 0.05). The data from Kaplan-Meier plotter showed a positive relationship between *BTG3* expression and the overall survival rate of the ovarian cancer patients with paclitaxel treatment (Figure 4D, *p* < 0.05). A positive correlation between BTG3 expression and the post-progression survival rate was observed in all ovarian cancer patients, or G_1-3_, G _2-3_, Grade_3_ or suboptimal cancer patients (Figure 4D, *p* < 0.05).

### The *BTG3*-related genes and pathways in cancers

**FIGURE 5:**
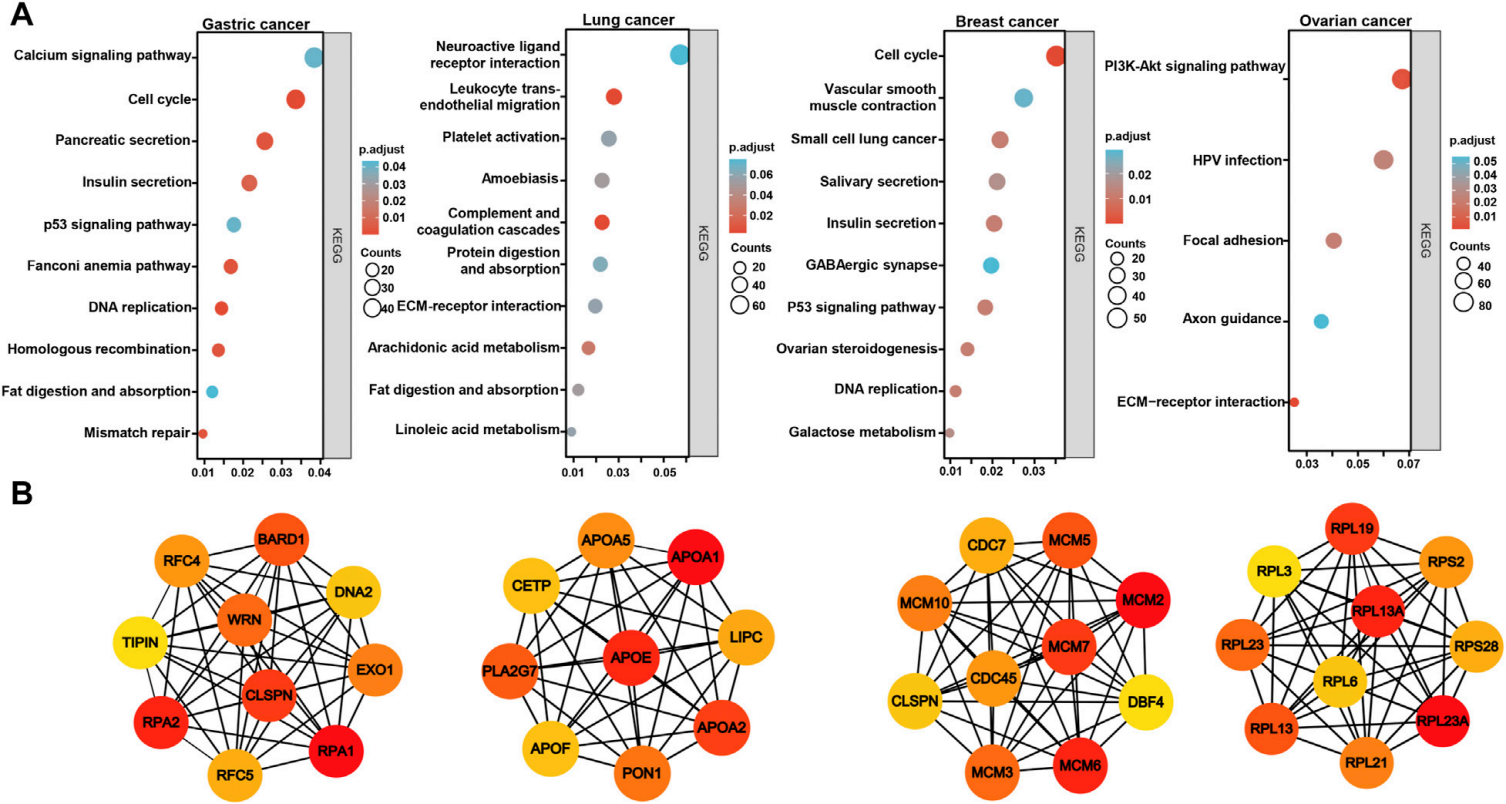
The differential genes and related signal pathways about *BTG3* in cancersn The differential genes of *BTG3* were subjected to the signal pathway analysis using KEGG **(A)**. STRING was used to identify the protein-protein interaction network of differential genes about *BTG3* in cancers, and Cytoscape was employed to find out the top 10 hub nodes ranked by degree **(B)**.

On the Xiantao platform, we found the differential genes between low and high expression groups of *BTG3* mRNA in cancers. KEGG analysis showed that the top signal pathways of the differential genes included cell cycle, calcium and p53 signal pathway, pancreatic and insulin secretion, fat digestion and absorption, DNA replication, mismatch repair and homologous recombination in gastric cancer, platelet activation and coagulation, digestion and absorption of protein and fat, metabolism of arachidonic and linoleic acids in lung cancer, cell cycle, salivary and insulin secretion, DNA replication, and ovarian steroidogenesis in breast cancer, and PI3K/Akt signal pathway, focal adhesion and ECM-receptor interaction in ovarian cancer (Figure 5A). In addition, the STRING was used to identify the PPI pairs and the cytoscape to find out the top 10 nodes ranked by degree (Figure 5B). The top hub genes mainly contained replication protein, DNA replication helicase, replication factor, WRN RecQ like helicase and exonucleases in gastric cancer, Apolipoproteins, lipase C, phospholipase A2, and cholesteryl ester transfer protein in lung cancer, minichromosome maintenance protein in breast cancer, and ribosomal proteins in ovarian cancer.

**FIGURE 6:**
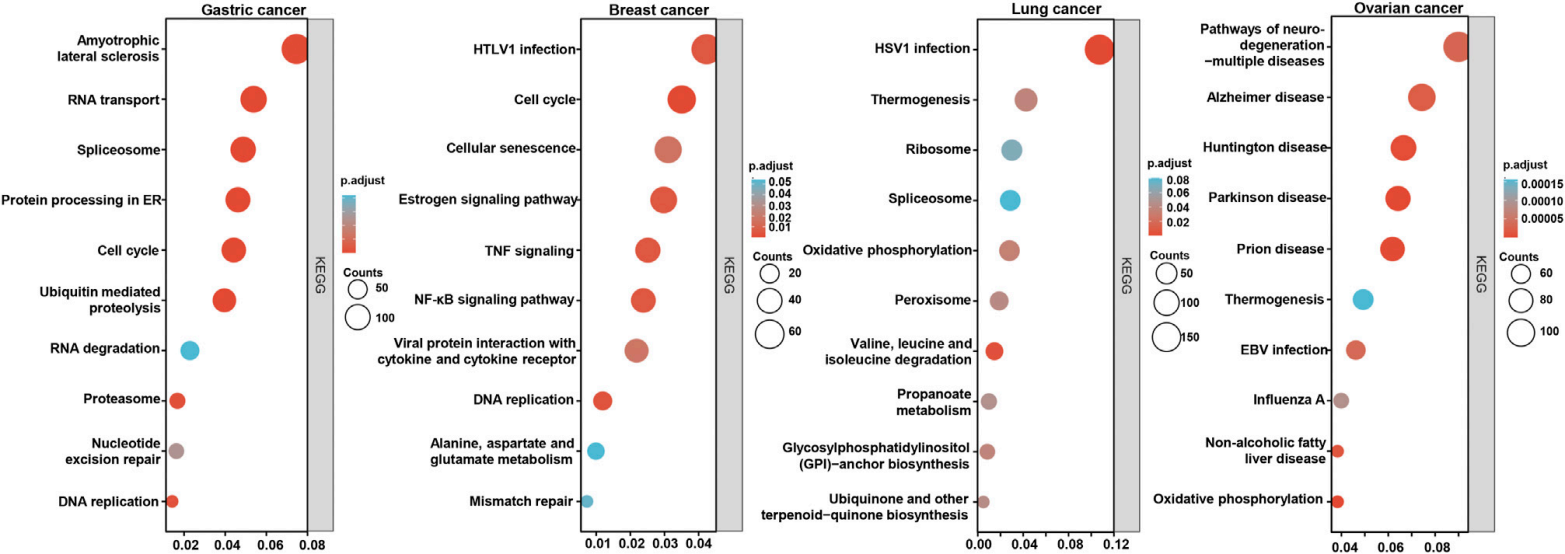
The *BTG3*-related signal pathways in cancers The top *BTG3*-related genes were screened and classified into the signal pathways of KEGG using Xiantao database.

According to the Xiantao database, the *BTG3*-correlated genes in cancers were analyzed and subjected to the KEGG analysis (Figure 6). The *BTG3*-correlated genes were involved in RNA transport, splicing and degradation, DNA replication and cell cycle, proteasomal degradation for gastric cancer, cell cycle, DNA replication, and mismatch repair, TNF and NF-κB signal pathways for lung cancer, ribosome and spliceosome, and metabolism of amino acids for breast cancer, neural diseases, viral infection, oxidative phosphorylation for ovarian cancer.

## Discussion

*BTG3* overexpression suppressed the proliferation and invasion of epithelial ovarian and colorectal cancer cells by weakening Akt/GSK3β/β-catenin signaling (Mao et al., 2016; An et al., 2017). Lv et al. (2018) found that *BTG3* knockdown promoted cell proliferation, migration, invasion, relieved G_2_ arrest, and inhibited apoptosis in colorectal cancer cells with PAK2 (p21 activated kinase 2), RPS6KA5 (ribosomal protein S6 kinase A5), YWHAB (tyrosine 3-monooxygenase/tryptophan 5-monooxygenase activation protein beta), and STAT3 up-regulated and RAP1A (ras-related protein rap-1A), DUSP6 (dual specificity phosphatase 6), and STAT (signal transducer and activator of transcription) 1 down-regulated. Our group demonstrated that *BTG3* expression inhibited proliferation, tumor growth, migration and invasion, and induced autophagy, apoptosis, and chemosensitivity to cisplatin, MG132 (proteasome inhibitor), paclitaxel, and SAHA (histone deacetylase inhibitor) in gastric and colorectal cancer cells (Gou et al., 2015; Zheng et al., 2017). In esophageal adenocarcinoma cells, *BTG3* upregulation suppressed the proliferation and invasion (Du et al., 2015). Lv et al. (2013) found that *BTG3* suppressed proliferation, invasion and induced G_1_/S cycle arrest of hepatocellular carcinoma cells. Reportedly, iASPP promoted epithelial-mesenchymal transition, and conferred cisplatin resistance in cervical cancer *via* miR-20a- FBXL5/BTG3 signaling (Xiong et al., 2017). Yanagida et al. (2013) demonstrated that the antisense transcript of *BTG3* gene (ASBEL) down-regulated *BTG3* protein and promoted proliferation and tumorigenicity of ovarian clear cell carcinoma. ASBEL knockdown functioned as a tumor suppressive role in breast cancer cells by up-regulating *BTG3* (Xia et al., 2017). *BTG3* anti-sense transcript mediated the down-regulation of ATF3 expression, which was essential for the proliferation and tumorigenicity of colon cancer cells (Taniue et al., 2016). In combination with these findings, it was suggested that *BTG3* might be employed as a molecular target of cancer gene therapy because of its inhibitory effects on aggressive phenotypes of cancer cells.

A body of evidence indicates that *BTG3* expression is down-regulated in gastric cancer (Gou et al., 2015; Ren et al., 2015), esophageal adenocarcinoma (Du et al., 2015), hepatocellular carcinoma (Lv et al., 2013), lung cancer (Chen et al., 2013), ovarian cancer (Deng et al., 2013), renal cell carcinoma (Majid et al., 2009), and colorectal cancer (Xiong et al., 2017) due to its promoter methylation (Yu et al., 2008; Majid et al., 2009; Lv et al., 2013; Gou et al., 2015), in line with our findings about lung and breast cancers. Additionally, *BTG3* methylation was negatively correlated with its mRNA expression, and was higher in cancer than in normal tissues of the stomach, lung, and breast. In breast and gastric cancer, the correlation between *BTG3* mRNA and aggressive behaviors was the opposite to that between *BTG3* methylation and them. These findings suggested that *BTG3* hypoexpression might be due to its promoter methylation. In contrast, our results showed *BTG3* mRNA overexpression in gastric and ovarian cancers. The controversial findings might be explained by different approaches: previous reports from immunohistochemistry, Western blot or RT-PCR, but the present study from the cDNA chip or transcriptomics.

In addition, BTG3 mRNA expression was positively linked to the differentiation of gastric cancer, in line with our previous report about gastric cancer tissues (Gou et al., 2015). *BTG3* was also reported to induce the differentiation of gastric and colorectal cancer cells, evidenced by a higher level of alkaline phosphatase (Gou et al., 2015; Zheng et al., 2017). These findings suggested that *BTG3* mRNA expression might underlie the molecular mechanisms of gastric cancer differentiation. In addition, *BTG3* expression was found to be negatively associated with the tumor size of lung cancer. Pulmonary adenocarcinoma patients had a higher *BTG3* mRNA expression than squamous cell carcinoma patients. A higher *BTG3* mRNA expression was seen in invasive ductal than lobular carcinomas and negatively correlated with N staging and favorable molecular subtypes (Luminal-type), suggesting that *BTG3* might be involved in the progression of breast cancer, and underlay the mechanisms of molecular subtyping. These results suggested that *BTG3* mRNA was employed to indicate the aggressive behaviors of lung cancer, and histogenesis of lung and breast cancers.

The prognostic significance of *BTG3* expression was analyzed, but controversial. Ren et al. (2015) found that *BTG3* expression was positively correlated with distant metastasis of gastric cancer, and the cancer patients with lower *BTG3* expression had a shorter overall survival time. Deng et al. (2013) demonstrated that *BTG3* expression was negatively associated with a higher incidence of metastasis, a better differentiation, longer disease-free time, and overall survival time of epithelial ovarian cancer as an independent factor of prognosis. However, there was no relationship between *BTG3* protein expression and overall survival rates of the patients with gastric (Gou et al., 2015) or colorectal (Zheng et al., 2017) cancer. In the present study, the positive correlation between *BTG3* mRNA expression and survival rate was seen in gastric and breast cancer patients, but not for lung and ovarian cancers according to Kaplan-Meier plotter. TCGA database showed that *BTG3* mRNA expression was an independent factor for favorable overall survival of lung cancer patients. *BTG3* mRNA was documented to indicate the adverse prognosis for pediatric T-cell acute lymphoblastic leukemia (Gottardo et al., 2007). Therefore, we concluded that the prognostic significance of *BTG3* expression was dependent on cancer type, pathological grouping, distinct methodologies, and different databases. Therefore, it should be careful to employ *BTG3* mRNA as a prognostic marker in clinicopathological practice.

KEGG analysis demonstrated that the *BTG3*-related pathways included cell cycle, pancreatic and insulin secretion, fat digestion and absorption, DNA replication, mismatch repair and homologous recombination in gastric cancer, platelet activation and coagulation, digestion and absorption of protein and fat, metabolism of arachidonic and linoleic acids in lung cancer, cell cycle, salivary and insulin secretion, and DNA replication in breast cancer. The top hub genes mainly contained replication protein and related enzymes in gastric cancer, the key enzymes and proteins for fat metabolism in lung cancer, minichromosome maintenance protein in breast cancer, and ribosomal proteins in ovarian cancer. Therefore, we speculate that *BTG3* might play important role in the cell cycle, fat digestion and metabolism, and protein biosynthesis, which be deeply investigated in the future.

In summary, *BTG3* mRNA might underlie the molecular mechanisms of the histogenesis of gastric, lung, and breast cancers. The paradoxical results about the prognostic significances of *BTG3* mRNA might result from tissue specificity, distinct grouping, and different data sources.

## Data Availability

The datasets presented in this study can be found in online repositories. The names of the repository/repositories and accession number(s) can be found in the article/Supplementary Material.
